# GSK-3β manipulates ferroptosis sensitivity by dominating iron homeostasis

**DOI:** 10.1038/s41420-021-00726-3

**Published:** 2021-11-03

**Authors:** Lingjuan Wang, Sijin Ouyang, Bin Li, Hao Wu, Fengli Wang

**Affiliations:** 1 Institute of Reproductive Health, Tongji Medical College, Huazhong University of Science and Technology, 430030 Wuhan, China; 2 Tianjin Medical University General Hospital, 300211 Tianjin, China; 3 State Key Laboratory of Agricultural Microbiology, College of Veterinary Medicine, Huazhong Agricultural University, 430070 Wuhan, China

**Keywords:** Cell death, Cancer therapeutic resistance

## Abstract

Ferroptosis is a newly characterized form of non-apoptotic-programmed cell death, which is driven by the lethal accumulation of iron-catalyzed lipid peroxides. Uncontrolled ferroptosis is implicated in the pathogenesis of a group of human diseases, while targeted induction of ferroptosis provides a potent therapeutic design for cancers. During the past decade, the fundamental regulatory circuits of ferroptosis have been identified. In this study, we show that the multifaceted Ser/Thr protein kinase GSK-3β acts as a positive modulator of the ferroptosis program. Pharmacological inhibition of GSK-3β by selective inhibitor LY2090314 or genetic KD of GSK-3β by shRNA potently promotes ferroptotic resistance. GSK-3β KD antagonizes the expression of iron metabolic components including DMT1, FTH1, and FTL, leading to the disruption of iron homeostasis and decline in intracellular labile free iron level. Taken together, our findings elaborate an indispensable role of GSK-3β in determining ferroptotic sensitivity by dominating cellular iron metabolism, which provides further insight into GSK-3β as a target for cancer chemotherapy.

## Introduction

Ferroptosis is a newly characterized form of regulated cell death [1]. This iron-dependent cell death is sharply distinct from the previously defined programmed cell death including apoptosis, necroptosis, and pyroptosis, at morphological, genetic, and biochemical levels. Glutathione peroxidase 4 (4GPX4) is the sole glutathione peroxidase that detoxifies the phospholipid hydroperoxides by utilizing reduced glutathione (GSH) as an essential cofactor [2]. Erastin, the canonical ferroptosis inducer, targets and restrains the cystine/glutamate antiporter system xc^−^, leading to decelerated cysteine uptake, GSH exhaustion, and GPX4 inactivation. Additionally, ferroptosis suppressor protein 1 (FSP1) [3, 4] and dihydroorotate dehydrogenase (DHODH) [5] operate in parallel to GPX4 to antagonize lipid peroxidation and restrain ferroptosis. Reversely, the biochemical nature of ferroptosis is fundamentally defined by the lethal production of lipid peroxides [6]. Lipid peroxidation occurring in polyunsaturated fatty acids (PUFAs)-containing phospholipids is largely dependent on enzymatic reactions, although the exact mechanisms are less understood. Lipoxygenases (LOXs) are dioxygenases responsible for directly oxygenating PUFAs and PUFAs-containing lipids in biological membranes [7, 8]. Acyl-CoA synthetase long-chain family member 4 (ACSL4) and lysophosphatidylcholine acyltransferase 3 (LPCAT3) catalyze the PUFAs esterification for the subsequent incorporation into membrane phospholipids, shape the cellular lipid composition and define the ferroptotic sensitivity [9, 10]. Moreover, it has been recently reported that POR (cytochrome P450 oxidoreductase) and CYB5R1 (NADH-cytochrome b5 reductase 1) promote lipid peroxidation and facilitate ferroptosis [11, 12].

The intracellular labile free iron is indispensable for lipid peroxidation and ferroptosis execution. In mammals, transferrin receptor 1 (TFR1) imports the transferrin-bound iron from the extracellular environment through endocytosis, while divalent metal transporter 1 (DMT1) mediates the ferrous iron across the brush border of intestinal epithelial cells. The cytosolic iron is safely sequestered in ferritin nanocages or supports the bioactive labile iron pool [13]. Growing evidence has emerged that disturbed the cellular iron metabolism compromises ferroptosis sensitivity in multiple cell lines [14]. Specifically, immune depletion of transferrin in serum or genetic silence of TFR1 significantly inhibits ferroptotic cell death [15]. Additionally, genetic ablation of ferritin heavy chain (FTH1) specifically in heart or neurons aggregates ferroptosis associated hypertrophic cardiomyopathy [16] or traumatic brain injury [17], respectively. Although the exact mechanism of iron driving ferroptosis is less characterized, iron-catalyzed production of free alkoxyl phospholipid and phospholipid peroxyl radicals through the Fenton reaction is essential for ferroptotic program. Furthermore, iron sustains the enzymatic activities of the iron-containing proteins, including LOXs and POR, which may be critical for their catalyzation of lipid peroxidation.

Although dysregulated ferroptosis is implicated in the pathogenesis of diverse human diseases, targeted induction of ferroptosis provides a potent therapeutic design to cancers, especially to therapy-resistant cancers [18]. Multiple tumors associated proteins determine the ferroptotic sensitivity and resistance in the context of cancers. Specifically, the tumor suppressor P53 has been reported to enhance ferroptotic sensitivity by transcriptionally inhibiting the expression of solute carrier family 7 members 11 (SLC7A11, the catalytic subunit of system xc^−^) [19] or by facilitating the expressions of spermidine/spermine N1-acetyltransferase 1 (SAT1) [20] and glutaminase 2 (GLS2, a mitochondrial glutaminase essential for glutaminolysis to fuel tricarboxylic acid (TCA) cycle, mitochondrial respiration, and adenosine triphosphate (ATP) generation [15, 21]. Reversely, P53 was also reported to suppress ferroptosis in cancers by blocking dipeptidyl peptidase-4 (DPP4)-mediated lipid peroxidation in a transcription-independent manner [22], or transcriptionally upregulating the target gene *CDKN1A* (encoding p21) [23]. In addition, the tumor suppressor BRCA1-associated protein 1 (BAP1) is a nuclear de-ubiquitinating enzyme to dominate the epigenetic regulation of gene transcription. It has been recently reported that BAP1 facilitates lipid peroxidation and promotes ferroptosis through repressing SLC7A11 [24]. It is worth noting that metastasis-prone mesenchymal cancers, which are highly resistant to chemotherapy and targeted therapy, are sensitive to ferroptosis [25, 26].

The Ser/Thr protein kinase glycogen synthase kinase 3β (GSK-3β) is a multifunctional protein kinase involved in diverse cellular metabolic processes. Specifically, GSK-3β phosphorylates and facilitates the proteasomal degradation of beta-catenin, thus negatively dominating the Wnt/beta-catenin signaling pathway [27]. Besides, it has been widely documented that the genetic alterations in GSK-3β impinge on the tumorigenesis, tumor development, epithelial–mesenchymal transition, and cancer metastasis, as well as therapy resistance in multiple cancers [28]. In this study, we uncover an indispensable role of GSK-3β in ferroptosis regulation. Pharmacological inhibition or genetic knockdown (KD) of GSK-3β sharply antagonizes erastin-initiated ferroptosis in cancer cells. Mechanistically, GSK-3β depletion declines the intracellular labile free iron pool by disrupting the cellular iron metabolism. This study highlights a potent therapeutic design for cancers by targeting the GSK-3β–iron metabolism–ferroptosis axis.

## Results

### Inhibition of GSK-3β kinase activity significantly promotes resistance to ferroptotic cell death

**Fig. 1:**
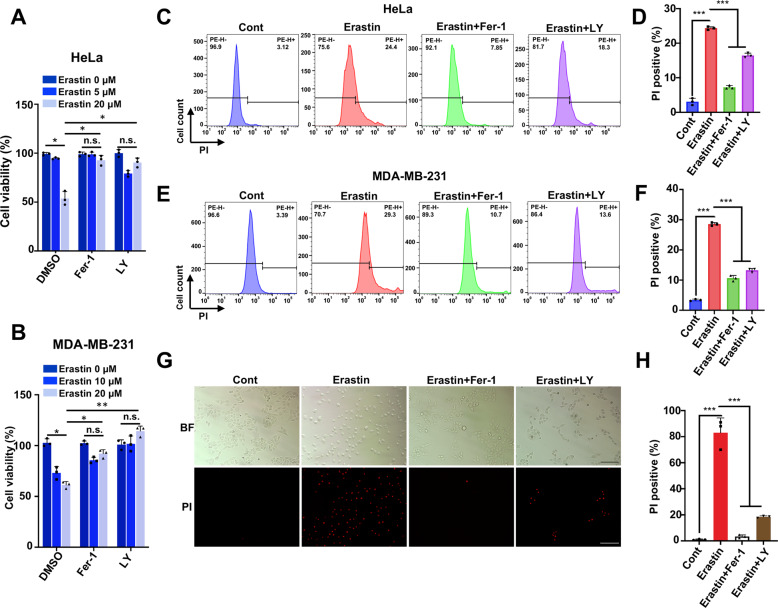
Inhibition of GSK-3β kinase activity promotes resistance to ferroptotic cell death. **A** Cell viability was assessed using CCK8 in indicated HeLa cells treated with erastin (0, 5, 20 μM) with or without ferrostatin-1 (Fer-1, 20 μM), with or without pretreated with GSK-3α/β inhibitor LY2090314 (LY, 20 nM) for 2 h prior to erastin treatment. **B** Indicated MDA-MB-231 cells were treated with erastin (0, 10, 20 μM) with or without ferrostatin-1 (Fer-1, 20 μM), or GSK-3α/β inhibitor LY (20 nM), respectively, and cell viability was assayed by CCK8. **C** Representative plotting data of HeLa cells from flow cytometry (FCM) analysis. Indicated HeLa cells were treated with erastin (35 μM), with or without Fer-1 (20 μM), with or without LY (20 nM) for 24 h, dead cells were determined as propidium iodide (PI) positive cells detected by FCM. **D** The percentage of PI-positive cell population in **C** was analyzed using FlowJo software (Version 10.0). **E** Representative plotting data of MDA-MB-231 cells from flow cytometry (FCM) analysis. Indicated MDA-MB-231 cells were treated with erastin (40 μM), with or without Fer-1 (20 μM), or LY (20 nM) for 24 h, dead cells were determined as propidium iodide (PI) positive cells detected by flow cytometry. **F** The percentage of PI-positive cell population in **E** was analyzed using FlowJo software (Version 10.0). **G** Fluorescence microscopy images with PI staining (in red) reflecting cell death after erastin treatment for HeLa cells at 35 μM combined with or without Fer-1(20 μM) and LY (20 nM). Upper, bright field (BF); Down, PI signal (PI). Scale bar, 200 μm. **H** The cell death (PI positive cells) in **G** was quantified. Data shown represent mean ± SD from three independent experiments. Shown above is representative image from three independent replicates. Statistical analysis was made using Student’s *t*-test; *p < 0.05, **p < 0.01, ***p < 0.001.

To gain insight into mechanisms of protein phosphorylation in modulating ferroptosis, we employed specific kinase inhibitors to identify the potential regulators that are essential for erastin-induced cell death. We paid attention to LY2090314 (hereafter referred to as LY), an effective and competitive GSK-3α/β inhibitor (IC_50_: 1.5 nM/0.9 nM), with little additional non-specific activity against other kinases [29]. To determine its involvement in erastin-induced ferroptosis process, a CCK8 assay was performed first to assess cell survival with presence or absence of LY along with erastin in HeLa cells. Notably, we observed that LY treatment significantly reversed erastin-induced cell death, like the suppression effect on cell survival by ferrostatin-1 (Fer-1), a specific inhibitor of ferroptosis [30] (Fig. 1A). In parallel, ferroptosis resistance caused by LY was also observed in MDA-MB-231 cells (Fig. 1B). We further evaluated the effect of GSK3 inhibitor on ferroptosis using PI staining followed by fluorescence microscopy or flow cytometry (FCM). PI fluorescence signal detected by FCM clearly showed an increased percentage of dead cells upon erastin treatment, whereas, co-treatment with LY or ferroptosis inhibitor Fer-1 resulted in a strong reduction in both HeLa and MDA-MB-231 cells (Fig. 1C–F). Similarly, PI-positive cells visualized by microscopy were much less after co-treatment with LY or Fer-1 compared with erastin treatment alone in HeLa cells (Fig. 1G, H). Moreover, depletion of GSH, the upstream ferroptotic event induced by erastin, was not affected by LY treatment, which suggested that the target of GSK3 in ferroptosis process was downstream of GSH depletion (Supplementary Fig. 1A, B). Collectively, these data demonstrated that inhibition of GSK-3β decreased the ferroptotic cell death induced by erastin in HeLa and MDA-MB-231 cells, and thus, indicated GSK-3β could play a role to promote ferroptosis sensitivity.

### GSK-3β is an indispensable positive ferroptosis regulator

**Fig. 2:**
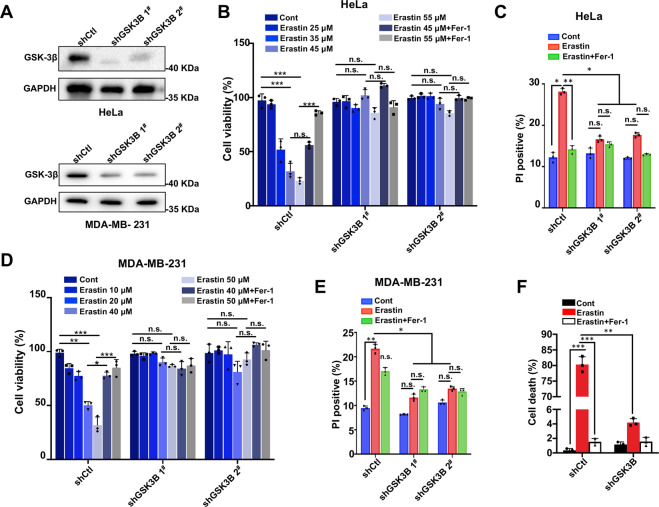
GSK-3β knockdown suppresses erastin-induced ferroptosis. **A** Western blot analysis of GSK-3β expression indicated GSK-3β knockdown (KD) in HeLa and MDA-MB-231cells using two specific short hairpin RNAs (shRNAs) (referred to shGSK-3β 1^#^ and 2^#^). Independent experiments are repeated three times and the shown is a representative image. **B** Indicated HeLa cells were treated with erastin (0, 25, 35, 45, and 55 μM) in combination with or without 20 μM Fer-1 for 24 h, cell viability was assayed using CCK8 kit. **C** Indicated HeLa cells were treated with erastin (35 μM) alone or with Fer-1 (20 μM) for 24 h, and cell death was measured by propidium iodide (PI) staining detected by flow cytometry. The percentage of the PI-positive cell population was analyzed using FlowJo software (Version 10.0). **D** Indicated MDA-MB-231 cells were treated with erastin (0, 10, 20, 40, and 50 μM) in combination with or without 20 μM Fer-1 for 24 h, and cell viability was assayed using a CCK8 kit. **E** Indicated MDA-MB-231 cells were treated with erastin (40 μM) alone or with Fer-1 (20 μM) for 24 h, and cell death was measured by propidium iodide (PI) staining detected by flow cytometry. The percentage of PI-positive cell population was analyzed using FlowJo software (Version 10.0). **F** Indicated HeLa cells were treated with erastin (35 μM) alone or with Fer-1 (20 μM), and cell death was measured by propidium iodide (PI) staining using fluorescence microscopy. The percentage of PI-positive (red) cells were quantitated. Data shown represent mean ± SD from three independent experiments. Statistical analysis was made using Student’s *t*-test; *p < 0.05, **p < 0.01, ***p < 0.001.

The above data suggest that GSK-3β is likely a novel positive regulator of ferroptosis. To further investigate the effect of GSK-3β on ferroptosis, we established two stable GSK-3β KD cancer cell lines (HeLa and MDA-MB-231) by using two specific short hairpin RNAs (shRNAs) (referred to as GSK-3β KD 1^#^ and 2^#^) (Fig. 2A). Using CCK8 assay, we observed that depletion of GSK-3β by shRNA significantly suppressed erastin-induced cell death in both HeLa and MDA-MB-231 cells (Fig. 2B, D). In line with CCK8 results, we observed remarkably decreased cell death after erastin induction in GSK-3β KD cells compared with control as determined by flow cytometry using PI staining (Fig. 2C, E and Supplementary Fig. 2A, B). Further, PI fluorescence signal visualized by microscope clearly displayed an increase of PI-positive cells after erastin treatment, which can be nearly completely blocked by Fer-1, whereas dramatically decreased in GSK-3β KD cells (Fig. 2F, Supplementary Fig. 2C). Thus, our results revealed that loss of GSK-3β conferred cells resistance to erastin-triggered ferroptotic cell death, suggesting a positive modulatory role of GSK-3β.

**Fig. 3:**
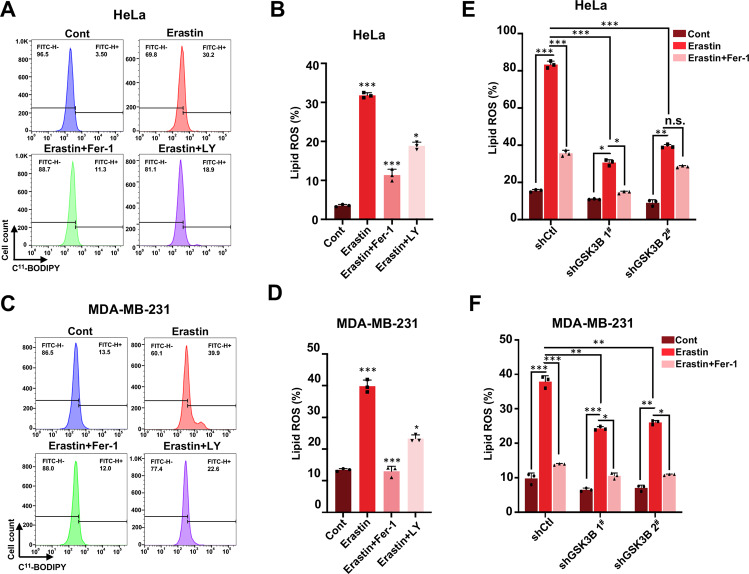
GSK-3β inhibition or GSK-3β knockdown affects the accumulation of lipid ROS. **A** Representative plotting data of HeLa cells through flow cytometry (FCM) analysis. Indicated HeLa cells were treated with erastin (35 μM) with or without Fer-1 (20 μM) or LY (20 nM) for 24 h, and lipid ROS production was detected by FCM after incubation with fluorescent probe C^11^-BODIPY. **B** Statistical analysis of the percentage of C^11^-BODIPY positive cells in **A** from three independent experiments. **C** Representative plotting data of MDA-MB-231 cells from flow cytometry (FCM) analysis. Indicated MDA-MB-231 cells were treated with erastin (45 μM) with or without Fer-1 (20 μM) or LY (20 nM) for 24 h, and lipid ROS production was detected by FCM using C^11^-BODIPY probe. **D** Statistical analysis of the percentage of C^11^-BODIPY positive cells in **C** from three independent experiments. **E** Indicated shCtl, shGSK-3β 1^#,^ and 2^#^ HeLa cells were treated with erastin (35 μM) with or without Fer-1 (20 μM) for 24 h, lipid ROS production was assayed by flow cytometry using C^11^-BODIPY. Statistical analysis of percentage of C^11^-BODIPY positive cells was performed from three independent experiments. **F** Indicated shCtl, shGSK-3β 1^#^ and 2^#^ MDA-MB-231 cells were treated with erastin (40 μM) with or without Fer-1(20 μM) for 24 h, and lipid ROS production was assayed by flow cytometry using C^11^-BODIPY. The numbers of BODIPY positive cells were quantitated. Data shown represent mean ± SD from three independent experiments. Statistical analysis was made using Student’s *t*-test; *p < 0.05, **p < 0.01, ***p < 0.001.

We next examined whether GSK-3β inhibition or GSK-3β KD affect accumulation of lipid reactive oxygen species (ROS), a classic erastin-induced ferroptotic event. Upon erastin treatment, elevated lipid ROS production detected by flow cytometry using the fluorescent probe C^11^-BODIPY was observed in control cells, however, GSK-3β inhibition by LY remarkably reduced accumulation of lipid ROS in both HeLa and MDA-MB-231 cells, as well as Fer-1 treatment (Fig. 3A–D). Moreover, we assessed lipid ROS production either in the presence or absence of Fer-1 in control and GSK-3β KD cells. GSK-3β depletion significantly impaired lipid ROS production induced by erastin in both HeLa and MDA-MB-231 cells (Fig. 3E, F and Supplementary Fig. 3A, B). In summary, under ferroptotic stimuli, GSK-3β inactivation by inhibitor or KD markedly weaken erastin-triggered ferroptotic effects including cell death and lipid ROS accumulation, which further strengthen the conclusion that GSK-3β can promote cell ferroptosis.

### GSK-3β preserves labile iron homeostasis during ferroptosis

**Fig. 4:**
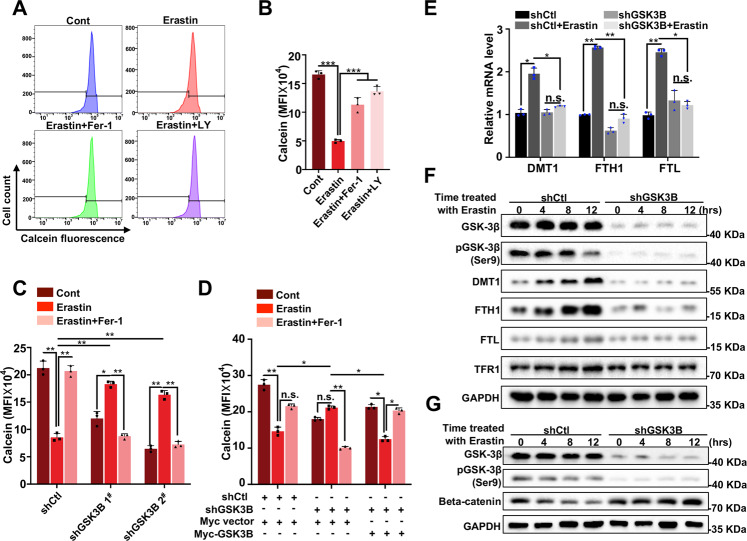
GSK-3β increases labile iron pool level and modulates iron metabolism-associated protein expression induced by erastin. **A** Indicated HeLa cells were treated with erastin (35 μM) with or without Fer-1 (20 μM) or LY (20 nM) for 24 h, and intracellular labile iron levels were determined by flow cytometry using Calcein-AM probe. **B** Quantification of mean fluorescence intensity (MFI) of calcein from **A** were shown. *Y-*axis represents MFI (×10^4^) and data show the mean ± SD from three independent experiments. **C** Indicated shCtl, shGSK-3β 1^#,^ and 2^#^ HeLa cells were treated with erastin (35 μM) with or without Fer-1 (20 μM) for 24 h, and intracellular labile iron level was assayed by flow cytometry using a calcein-AM probe. The mean fluorescence intensity (MFI) of calcein from each group was quantitated. *Y*-axis represents MFI (×10^4^) and data show the mean ± SD from three independent experiments. **D** Indicated shCtl or shGSK-3β HeLa cells were transfected with either a control plasmid (myc-vector) or myc-GSK-3β plasmid. Cells were treated with erastin (35 μM) with or without Fer-1 (20 μM) for 24 h, and intracellular labile iron level was assayed by flow cytometry using Calcein-AM probe. The mean fluorescence intensity (MFI) of calcein from each group was quantitated. *Y*-axis represents MFI (×10^4^) and data show the mean ± SD from three independent experiments. **E** Indicated shCtl or shGSK-3β HeLa cells were treated with erastin (35 μM) with or without Fer-1 (20 μM) for 24 h. The mRNA level of DMT1, FTH1, and FTL in indicated cells was assayed by RT-qPCR. The relative gene expression is normalized to GAPDH. Data shown represent mean ± SD from three independent experiments. **F** Indicated shCtl or shGSK-3β HeLa cells were treated with 35 μM of erastin for different times (0, 4, 8, 12 h) as indicated, and subjected to Western blotting for GSK-3β, pGSK-3β (Ser9), DMT1, FTH, FTL, and TFR1. **G** Indicated shCtl or shGSK-3β HeLa cells were treated with 35 μM of erastin for different times (0, 4, 8, 12 h) as indicated, and subjected to Western blotting for GSK-3β, pGSK-3β (Ser9), and beta-catenin. Statistical analysis was made using Student’s *t*-test; *p < 0.05, **p < 0.01, ***p < 0.001.

Given that GSK-3β KD induces cell insensitivity to erastin, we next elucidated the potential underlying mechanism of ferroptosis resistance. Intracellular iron is indispensable for accumulating lipid peroxides and the execution of ferroptosis. Thus, iron import, transport and storage, and turnover contribute to ferroptosis sensitivity. Accordingly, we sought to determine whether GSK-3β would affect levels of labile iron upon ferroptosis induction. Accumulation of intracellular free iron under ferroptotic conditions was evaluated using Calcein-AM probe labeling followed by flow cytometry detection. As expected, erastin treatment triggered an obvious decreased mean fluorescence intensity (MFI) of Calcein, which indicated elevated free iron level, whereas, GSK-3β inactivation by LY impaired the accumulation of cytosolic iron in response to ferroptotic stimuli (Fig. 4A, B). Consistently, GSK-3β silencing showed apparently increase of Calcein MFI after erastin treatment, which suggested the reduction in labile iron level (Fig. 4C, Supplementary Fig. 4A). And further, re-introduce of GSK-3β protein by transfected GSK-3β-expressing vector significantly rescued cellular free iron level compared with those in KD cells transfected with empty vector, which confirmed its indispensable role for iron maintenance (Fig. 4D, Supplementary Fig. 4B). The above observations demonstrated that GSK-3β inhibition or KD limited the intracellular accumulation of free iron and thus inhibited the onset of ferroptosis.

Intracellular labile iron level is controlled by a cellular iron homeostatic network of which iron can be uptaken into cells via transferrin receptor on the plasma membrane, transported by DMT1, and stored through binding ferritin [31]. To investigate the mechanistic link between GSK-3β and the labile iron level, we decided to determine whether GSK-3β KD would affect the expression levels of key cellular iron metabolism regulators. As shown in Fig. 4E, the mRNA levels of genes coding DMT1, FTH1, and light chain (FTL) are significantly increased in response to erastin, however, GSK-3β depletion abolished upregulation of these genes under erastin treatment condition (Fig. 4E). Accordingly, the protein levels of DMT1, FTH1, FTL, and TFR1 gradually elevated in a time-dependent manner following erastin treatment, KD of GSK-3β dramatically impaired the increase of DMT1, FTH1, FTL, with a slight impact on TFR1 protein level (Fig. 4F).

GSK-3β is a central regulator of the canonical Wnt signal transduction pathway through which modulates the expression of downstream genes. It forms a complex with axin, adenomatous polyposis coli (APC) to mediate beta-catenin phosphorylation, which is required for beta-catenin ubiquitination and degradation. Upon activation of the receptor Frizzled by Wnt ligand, phosphorylation of beta-catenin is blocked to bypass the degradation, then translocates into the nucleus to function as transcription co-factor [32]. We asked whether the expression of iron homeostasis maintenance factors was regulated by GSK-3β via beta-catenin level modulation. As shown in Fig. 4G, under erastin treatment conditions in control cells, GSK-3β was activated indicated by the decreased phosphorylation of Serine 9, which was the inhibitory modification of GSK-3β. As expected, gradually loss of beta-catenin was observed in control cells due to GSK-3β activation, and conversely significantly accumulated in GSK-3β KD cells, which suggested that GSK-3β depletion stabilized beta-catenin in response to erastin (Fig. 4G). Taken together, these data suggested that GSK-3β KD downregulated DMT1, FTH1, FTL expression and upregulated beta-catenin level, therefore prevented intracellular accumulation of free iron and promoted ferroptosis resistance.

### Re-expression of GSK-3β rescues the phenotypes induced by GSK-3β KD

**Fig. 5:**
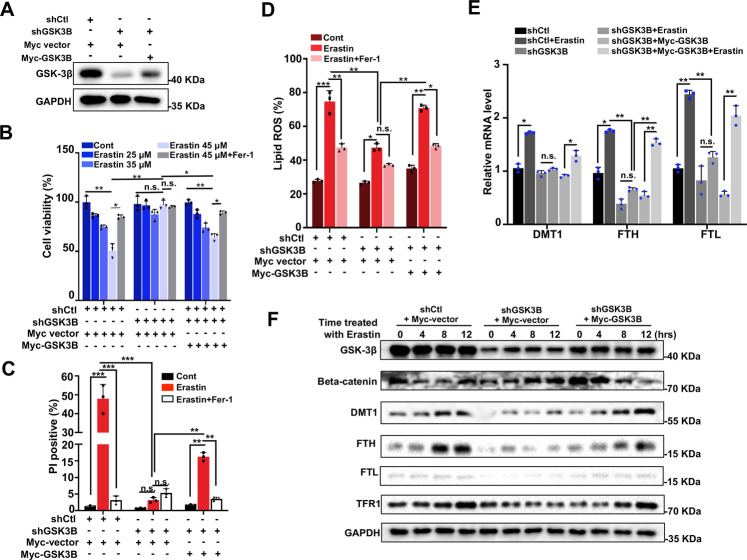
Re-expression of GSK-3β restores GSK-3β depletion-resisted ferroptosis. **A** Indicated shCtl or shGSK-3β HeLa cells were transfected with either control plasmid (myc-vector) or myc-GSK-3β plasmid. Re-expression of GSK-3β was confirmed by immunoblotting. **B** shCtl or shGSK-3β HeLa cells transfected with myc-vector or myc-GSK-3β were treated with erastin (35 μM) with or without Fer-1 (20 μM) for 24 h, cell viability was assayed using a CCK8 kit. **C** shCtl or shGSK-3β HeLa cells transfected with myc-vector or myc-GSK-3β were treated with erastin (35 μM) with or without Fer-1 (20 μM) for 24 h, and cell death was measured by propidium iodide (PI) staining using fluorescence microscopy. The percentage of PI positive (red) cells were quantitated. Data shown represent mean ± SD from three independent experiments. **D** shCtl or shGSK-3β HeLa cells transfected with myc-vector or myc-GSK-3β were treated with erastin (35 μM) with or without Fer-1 (20 μM) for 24 h, and lipid ROS production was detected by flow cytometry using C^11^-BODIPY. The percentage of BODIPY-positive cells in **C** were quantitated. Data shown represent mean ± SD from three independent experiments. **E** shCtl or shGSK-3β HeLa cells transfected with myc-vector or myc-GSK-3β were treated with 35 μM erastin for 24 h, and the mRNA expression of DMT1, FTH1, and FTL in indicated HeLa cells was assayed by RT-qPCR. The relative gene expression is normalized to GAPDH. Data shown represent mean ± SD from three independent experiments. **F** Indicated shCtl, shGSK-3β, and shGSK-3β transfected with myc-GSK-3β HeLa cells were treated with 35 μM erastin for different times (0, 4, 8, 12 h) as indicated, and subjected to Western blotting for GSK-3β, pGSK-3β (Ser9), beta-catenin, DMT1, FTH, FTL, and TFR1. Statistical analysis was made using Student’s *t*-test; *p < 0.05, **p < 0.01, ***p < 0.001.

To better confirm the essential function of GSK-3β in cell ferroptosis, we determined whether ectopic expression of GSK-3β protein in KD cells could restore erastin-induced cell death. We generated GSK-3β-expression vector, then transfected it into stable GSK-3β KD cell line, and observed successful expression (Fig. 5A). The cell viability detected by CCK8 assay showed that cell death significantly enhanced in GSK-3β-expressing groups compared with those in KD groups after erastin treatment, which indicated that erastin resistance was caused by GSK-3β depletion was abolished by re-expression of GSK-3β (Fig. 5B). This suggested that the proper function of GSK-3β was required for the ferroptosis-promoting function. Accordingly, cell death determined by PI staining sharply increased after erastin treatment in GSK-3β re-expressing cells than those transfected by empty vector (Fig. 5C, Supplementary Fig. 5A). In line with cell death results, a strong rescue effect of lipid peroxidation levels was also observed in KD cells re-introducing GSK-3β compared with that of control and GSK-3β KD transfected with empty vector groups (Fig. 5D, Supplementary Fig. 5B).

To further evaluate the effect of GSK-3β on gene expression of iron homeostatic modulators, we examined the mRNA levels of well-characterized genes which modulated cellular iron levels. As shown in Fig. 5E, erastin triggered the upregulation of DMT1, FTL, and FTH1 gene expression, whereas GSK-3β KD eliminated the effect of erastin treatment. As expect, transfection of myc-GSK-3β but not the empty vector into GSK-3β KD cells restored DMT1, FTL, and FTH1 mRNA levels, confirming that GSK-3β is involved ferroptosis through gene expression regulation. In line with this, a significant increase in DMT1, FTL, FTH1, and TFR1 protein levels was observed in GSK-3β re-expressed cells under either basal or erastin treatment conditions. Degradation of beta-catenin in response to erastin can be also detected after re-introducing GSK-3β (Fig. 5F). Taken together, the above data further confirmed the function of GSK-3β in the ferroptosis process, which modulated the expression of iron homeostasis maintenance factors and beta-catenin level.

## Discussion

Targeted induction of ferroptosis provides a promising therapeutic drug design against cancers. During the past decade, growing studies have been devoted to discover novel regulators and dissect the underlying molecular mechanisms of ferroptosis process in the context of cancers. In the current study, we uncover a novel ferroptotic regulatory protein and show that GSK-3β kinase is indispensable for ferroptosis execution by determining the cellular labile free iron pool. GSK-3β is a highly conversed and ubiquitously expressed Ser/Thr protein kinase, which was initially discovered as a specific kinase toward glycogen synthase. GSK-3β is now known to regulate a wider range of metabolic pathways and gene expression through targeting over than 100 substrates [33]. It was reported that GSK-3β is phosphorylated and inactivated by multiple kinases including PKB/AKT [34] and S6K (70-kDa ribosomal S6 kinase) [35]. Recently, independent studies suggested that erastin treatment results in the inactivation of these upstream kinases in diverse types of cancer cell [36, 37]. In our study, GSK-3β kinase is activated as shown by the dephosphorylation at Ser9 residue and the degradation of its substrate beta-catenin during ferroptosis induction mediated by erastin exposure (Fig. 4F, G). However, the underlying mechanism for which GSK-3β kinase is responsive to erastin and ferroptotic signaling is still elusive. This study highlights a potential therapeutic design to cancers by targeting the GSK-3β and ferroptosis.

It has been documented that GSK-3β governs several cell death pathways. GSK-3β directly phosphorylates the B-cell lymphoma-2 (BCL-2) family member BCL-2-associated X protein (BAX) at Ser163 residue, and promotes the localization of BAX onto mitochondria for the release of Cytochrome-c and subsequent apoptotic program [38]. Additionally, GSK-3β phosphorylates the anti-apoptotic myeloid cell leukemia-1 (MCL-1) and facilitates its ubiquitination for proteasomal degradation, leading to mitochondrial outer membrane permeabilization and aggregation of apoptotic cell death [39, 40]. On contrary, growing evidence has emerged that GSK-3β is anti-apoptotic. Genetic disruption of GSK-3β gene in mice results in multifocal hemorrhagic degeneration in liver owing to the severe hepatocyte apoptosis, leading to embryonic lethality between E (embryonic days)13.5 and E14.5. The reduction in nuclear factor-κB (NF-κB)-mediated anti-apoptotic response to tumor necrosis factor alpha (TNF-α) is observed after GSK-3β ablation [41]. Moreover, Xie and colleagues reported that the phosphorylation of GSK-3β at Ser9 disturbs the formation of the receptor-interacting protein kinase 3 (RIPK3)–mixed lineage kinase domain-like protein (MLKL) complex and suppresses necroptosis, putting forward to the notion that GSK-3β is a novel pro-necroptotic molecule [42]. Besides, several other studies showed that GSK-3β is critical for the inflammasome activation and pyroptotic cell death [43–45]. In this study, we found that pharmacological inhibition or gene silencing of GSK-3β significantly antagonizes ferroptotic cell death in HeLa and MDA-MB-231 cancer cells (Figs. 1 and 2), thus highlighting the ferroptosis modulating capacity of GSK-3β and expanding a novel function of GSK-3β in manipulating cell death and survival. This is similar to a recent study. Wu and colleagues showed that silence of GSK-3β blocked erastin-induced ferroptosis, while ectopic expression of GSK-3β sensitized this ferroptosis [46].

To further explore the potential dysregulated ferroptosis-related molecules caused by GSK-3β inhibition, we determined the intracellular labile iron level, due to the indispensable role of free iron for lipid peroxidation and ferroptosis execution. Either GSK-3β inhibition or GSK-3β KD can decline the erastin-mediated elevation of labile-free iron pool, which could be restored by GSK-3β complementation (Fig. 4B–D). The intracellular labile iron pool is coordinated by iron metabolism including iron uptake, iron storage, iron export, and ferritin turnover. All of these iron metabolic processes have been illustrated to impinge in ferroptosis execution [14]. Here we show that GSK-3β dominates the expression of iron metabolism genes including iron uptake associated DMT1, as well as iron storage associated FTH1 and FTL during erastin challenge, both at protein and mRNA levels (Figs. 4E, F and 5E). However, it is still unknown whether these genes are downstream targets of GSK-3β signaling or not. Given that beta-catenin is a well-known transcriptional regulator downstream of GSK-3β signaling, it is proposed that DMT1, FTH1, and FTL could be regulated by GSK-3β/beta-catenin axis. Indeed, exposure of Wnt-3a, a representative ligand that activates the beta-catenin pathway, leads to a substantial transcriptional activation of TFR1 in murine mammary epithelial C57MG cells [47]. Additionally, activation of NF-E2-related factor 2 (NRF2) transcriptionally elevates FTH1 and FTL expression, while NRF2 knockout sharply decreases their expression [48]. It is thus supposed that GSK-3β may regulates the transcription though NRF2, as NRF2 was reported as a substrate of GSK-3β kinase [49]. It has been widely documented that iron and iron metabolism could finely tune the GSK-3β and its downstream Wnt signaling [50, 51]. Therefore, our study and these previous studies collectively suggest a backward regulation of cellular iron metabolism by GSK-3β kinase.

**Fig. 6:**
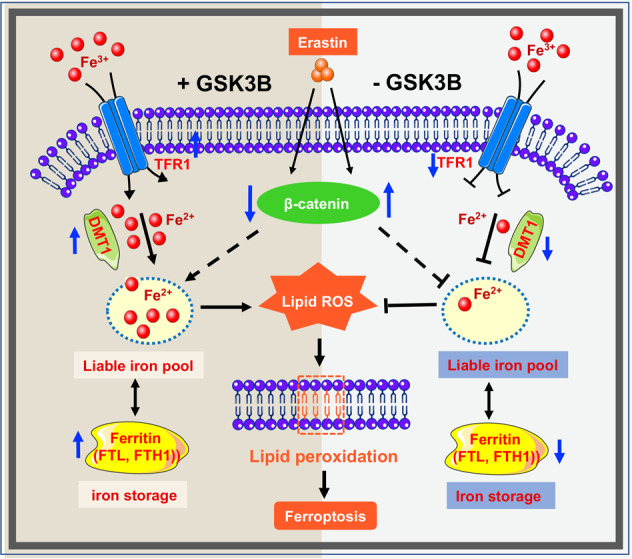
Schematic diagram deciphering the effect of GSK-3β on ferroptosis. In the presence of GSK-3β (left half), erastin activates degradation of beta-catenin and enhances the transcription of iron uptake, transportation, and storage genes (DMT1, FTH1, and FTL), which leads to an increase in cellular iron concentration. Iron overload results in lipid peroxidation and eventually exerts ferroptosis. In the absence of GSK-3β (right half), erastin-induced ferroptosis is restrained through beta-catenin elevation, and dysregulation of iron metabolism-associated protein (DMT1, FTH1, and FTL), thus cellular labile iron pool is depleted. Decreased intracellular total iron levels and lipid peroxidation further limits erastin-induced ferroptosis.

Taken together, we identified GSK-3β as a novel positive regulator of ferroptosis, which can be activated upon ferroptosis induction. GSK-3β may transcriptionally manipulate the expression of cellular iron metabolism regulators including TFR1, DMT1, FTH1, and FTL, of which dysregulation can lead to enhanced ferroptosis resistance (Fig. 6). Our findings expand the understanding of the mechanisms mediated by kinases involved in ferroptosis through intracellular iron level regulation.

## Materials and methods

### Cell culture, regents and plasmids

All cell lines (HeLa, MDA-MB-231, and HEK293T) were obtained from the American Type Culture Collection and were maintained in Dulbecco’s modified Eagle medium (DMEM, Gibco, 8121298) supplemented with 10% fetal bovine serum (FBS, Gibco, 2176398) and 1% penicillin–streptomycin at 37 °C with 5% CO_2_.

The following regents were used in this study: Erastin (E7781), Fer-1 (S7243), and LY2090314 (S7063) were purchased from Selleck (Houston, TX, USA). The coding region of GSK-3β gene was cloned into the pCMV vector in-frame with a myc tag within the vector. The pLKO.1-puro shRNA vector was obtained from Sigma-Aldrich (SHC001). The pLKO.1-shGSK-3β 1^#^ and 2^#^ were constructed using specific primer containing separate targeting sequence. (targeting sequence to GSK-3β 1^#^: 5′-CACTGGTCACGTTTGGAAAGA-3′, GSK-3β 2^#^: 5′-CCCAAACTACACAGAATTTAA-3′).

### RNA interference and construction of stable cell lines

Lentivirus was produced through transfecting psPAX2 (packaging vector), pMD2.G (envelope vector) and shRNA plasmids into HEK293T cells by PEI MAX (#24765, polysciences). After 48 h transfection, virus-containing medium was harvested.

To generate the stable KD cells, HeLa and MDA-MB-231 cells were infected with virus containing either pLKO.1-shGSK-3β 1^#^, pLKO.1-shGSK-3β 2^#^ or pLKO.1-control, and 1.5 mL medium was added with 1.5 μL polybrene (10 μg/mL, Sigma-Aldrich, H9268). After 48 h transfection, 1 μg/mL puromycin (Sigma-Aldrich, P9620) for HeLa cells or 3 μg/mL for MDA-MB-231 cells was added to the medium for selection. Stable KD cells were identified by western blotting to confirm GSK-3β KD efficiency after 5 days post infection.

### Cell viability assay

Cell viability was measured using Cell Counting Kit-8 (CCK-8, C0039, Beyotime, Shanghai, China). Briefly, HeLa and MDA-MB-231cells were seeded onto 96-well plates at a density of 8 × 10^3^/well. Next day, cells were treated with chemical regents (erastin, Fer-1, or LY2090314) at different concentrations for 24 h. Subsequently, cells were exposed to 100 μL cultural medium containing 10 μL CCK-8 reagent per well, and incubated for 1.5 h at 37 °C, 5% CO_2_ incubator. The absorbance was determined at a wavelength of 450 nm using a micro-plate reader (Synergy HTX, BioTek, USA).

### Cell death detection

Cell death was measured by propidium iodide (PI) staining followed by flow cytometry or fluorescence microscopy detection. Cells were seeded at a density of 70–80% confluence into 12-well plates and treated with erastin, Fer-1, and LY2090314. Next day, cells were incubated with 5 μg/mL PI (Sigma, P4170) for 15 min and observed by fluorescence microscopy. To detect the cell death using flow cytometry, cells were collected including floating dead cells, stained with 5 μg/mL PI, and the percentage of PI-positive cell population was analyzed by the flow cytometry. Minimum of 10,000 single cells were analyzed per well and all experiments were carried out at least in triplicate. The data analysis was performed using FlowJo software (Version 10.0).

### Lipid peroxidation detection

Cells were seeded into 12-well plates and cultured overnight. On the next day, cells were treated with chemicals for the indicated times. Then, cells were incubated with 10 μM of C^11^-BODIPY 581/591 (Invitrogen, D3861), then washed with PBS for three times. After 20 min, cells were harvested by trypsinization and resuspended in 300 μL PBS containing 10% FBS. To measure the lipid peroxidation, fluorescence from cells was assessed by flow cytometry (BD Biosciences) using a 488 nm laser on FL1 detector. Minimum 10,000 single cells were analyzed per well. The data was analyzed using FlowJo software (Version 10.0).

### Intracellular labile iron level measurement

Intracellular labile iron level was measured by Calcein-AM (ATT Bioquest, Inc., 22002) probe using flow cytometry (BD Accuri C6). The day before detection, cells were seeded into 12-well plates. The next day, cells were treated with chemicals (erastin, Fer-1, and LY2090314) for the indicated times, incubated with 5 μM of Calcein-AM for 20 min, then washed with PBS for three times. Cells harvested by trypsinization were resuspended in 300 μL PBS containing 10% FBS, and analyzed by flow cytometry, using a 488 nm laser on a FL1 detector. A minimum of 20,000 cells were examined of each condition. The data analysis was performed using FlowJo software (Version 10.0).

### Western blotting

Protein was extracted from cells using lysis buffer (20 mM Tris, 1% NP40, 10% glycerol, 137 mM NaCl, 2 mM EDTA, pH 7.2, 1 mM NaF, 1 mM PMSF, 1 mM Na_3_VO_4_). Proteins were separated by SDS–PAGE gel, and then transferred onto polyvinylidene difluoride membranes (PVDF, IPVH00010, Millipore, USA). The membranes were blocked with 5% fat-free milk for 1.5 h at room temperature, incubated with the primary antibodies overnight at 4 °C and subsequently incubated with HRP-conjugated secondary antibodies for 2 h at room temperature. Finally, proteins were visualized by enhanced chemiluminescence detection (US Everbright Inc, IS0527). The primary antibodies used were listed as follows: GSK-3β (Cell Signaling Technology, D5C5Z), p-GSK-3β (Ser9) (Cell Signaling Technology, 5558S), beta-catenin (Cell Signaling Technology, D10A8), FTH1 (Cell Signaling Technology, D1D4), FTL (ProteinTech, 10727-1-AP), DMT1 (ProteinTech, 20507-1-AP), TFR1 (ProteinTech, 66180-1-lg). GAPDH (ProteinTech, 60004-1) was used as a loading control for normalization.

### Total RNA extraction and RT-qPCR analysis

HeLa cells (both shRNA control and GSK-3β-KD) were treated with erastin (35 μM) for 12 h. Total RNA was extracted using TRIzol reagent (Invitrogen, 15596018, Carlsbad, CA, USA) and then used for cDNA synthesis (Yeasen, 11120ES60, China). Quantitative RT-PCR was performed on diluted cDNA with SYBR green Master Mix (Yeasen, 11203ES03, China) in a PCR thermocycler (StepOnePlus Real-time PCR system, Thermo Fisher Scientific, USA). All of primers used in this study were:

FTH1, forward 5′-TGAAGCTGCAGAACCAACGAGG-3′, reverse 5′-GCACACTCCATTGCATTCAGCC-3′; DMT1, forward 5′-AGCTCCACCATGACAGGAACCT-3′, reverse 5′-TGGCAATAGAGCGAGTCAGAACC-3′; FTL, forward 5′-TACGAGCGTCTCCTGAAGATGC-3′, reverse 5′-GGTTCAGCTTTTTCTCCAGGGC-3′.

Relative mRNA expression was determined by normalizing the cDNA quantity to that of GAPDH and calculated using the 2^−ΔΔCq^ method.

### GSH assay

Cells were seeded on 12-well plates and treated with either erastin, Fer-1 or LY2090314 for the indicated time. After treatment, cells were washed with ice-cold PBS and harvested by trypsin digestion. Then, harvested cells were deproteinated and centrifuged at 1500 rpm for 10 min. The samples were freeze-thawed twice by liquid nitrogen and 37 °C water bath. The total glutathlone and oxidized glutathione disulfide (GSSG) concentration in cell lysates was determined by the 5,5′-dithiobis (2-nitrobenzoic acid) (DTNB)-oxidized GSH reductase-recycling assay, according to the manufacturer’s instructions of the GSH Assay Kit (Beyotime, S0053, Shanghai, China). The absorbance of yellow product was monitored continuously at 412 nm with a microplate reader for 25 min. Standards (0.5, 1.0, 2.0, 5.0, 10.0, and 15.0 μM) of total glutathlone and GSSG were also assayed. All experiments were repeated three times, and values are shown as the mean ± SD.

### Statistical analysis

All experiments were conducted independently for three times with consistent results. Data were presented as mean ± SD and statistical significances were determined by a Student’s *t*-test. All statistical analyses were performed using *GraphPad Prism 8* software. *p* value < 0.05 (*p* < 0.05) was considered as statistically significant.

## Supplementary information

Supplementary Fig.1

Supplementary Fig.2

Supplementary Fig.3

Supplementary Fig.4

Supplementary Fig.5

Supplementary Figure legends

## Data Availability

All data generated or analyzed during this study are included in this published article (and its supplementary information files).
