# Supplementary figures and images for: GSK-3β manipulates ferroptosis sensitivity by dominating iron homeostasis
Source: Cell Death Discov. 2021 Nov 3;7:334. doi: 10.1038/s41420-021-00726-3 (PMC8566589; doi:10.1038/s41420-021-00726-3)

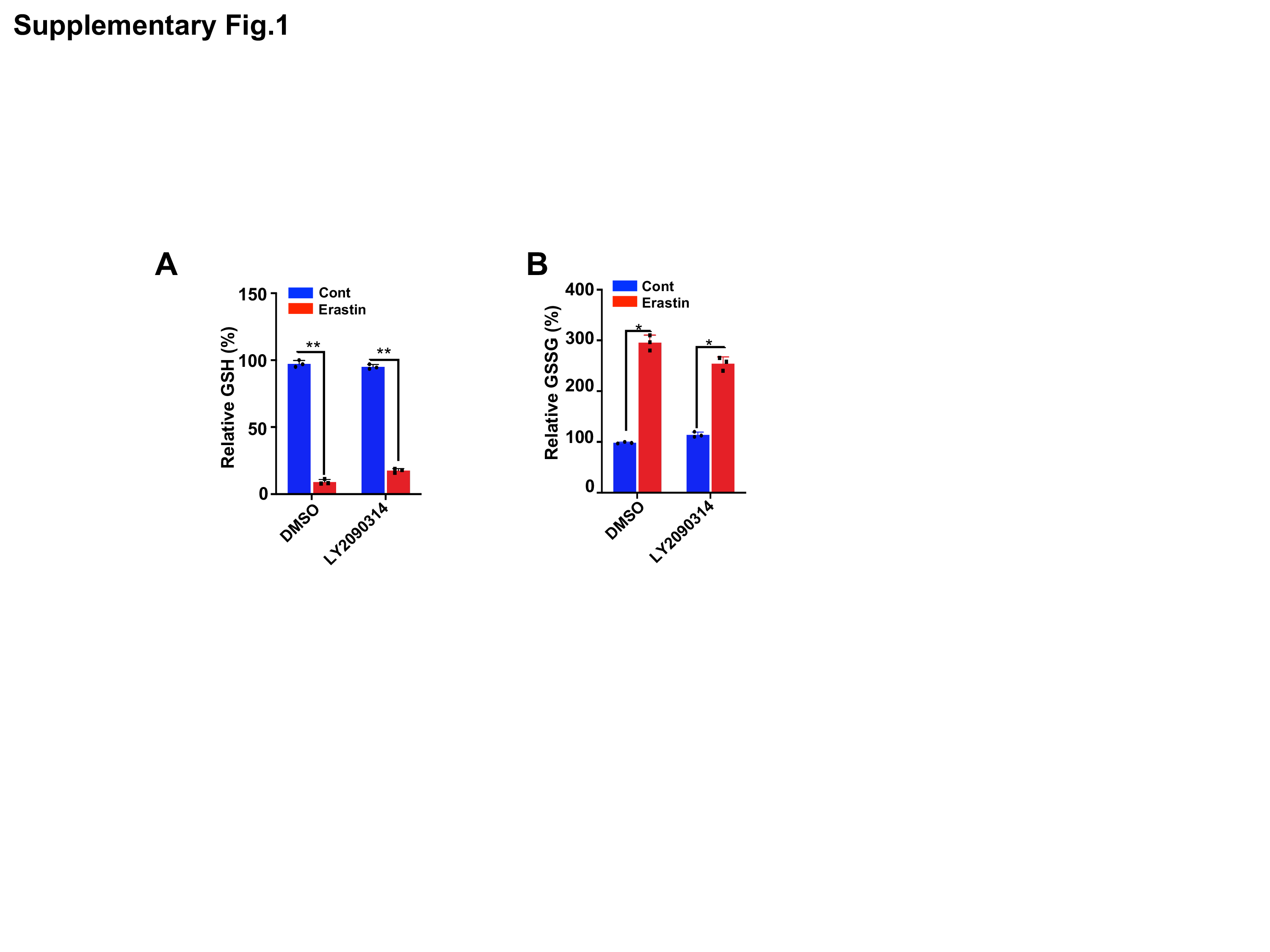

Supplement: Supplementary file 1 — Supplementary Fig.1 [file 41420_2021_726_MOESM1_ESM.tif]

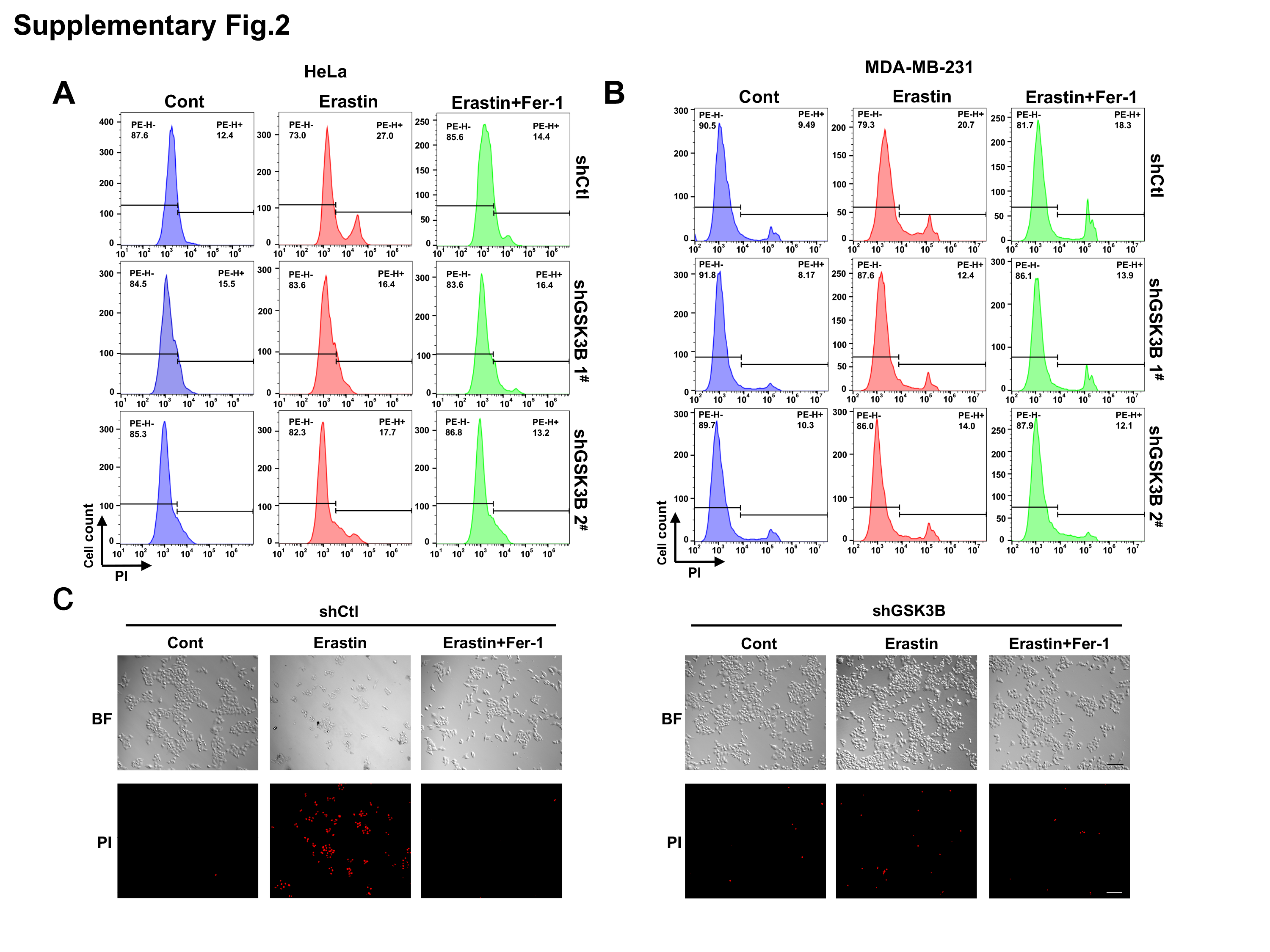

Supplement: Supplementary file 2 — Supplementary Fig.2 [file 41420_2021_726_MOESM2_ESM.tif]

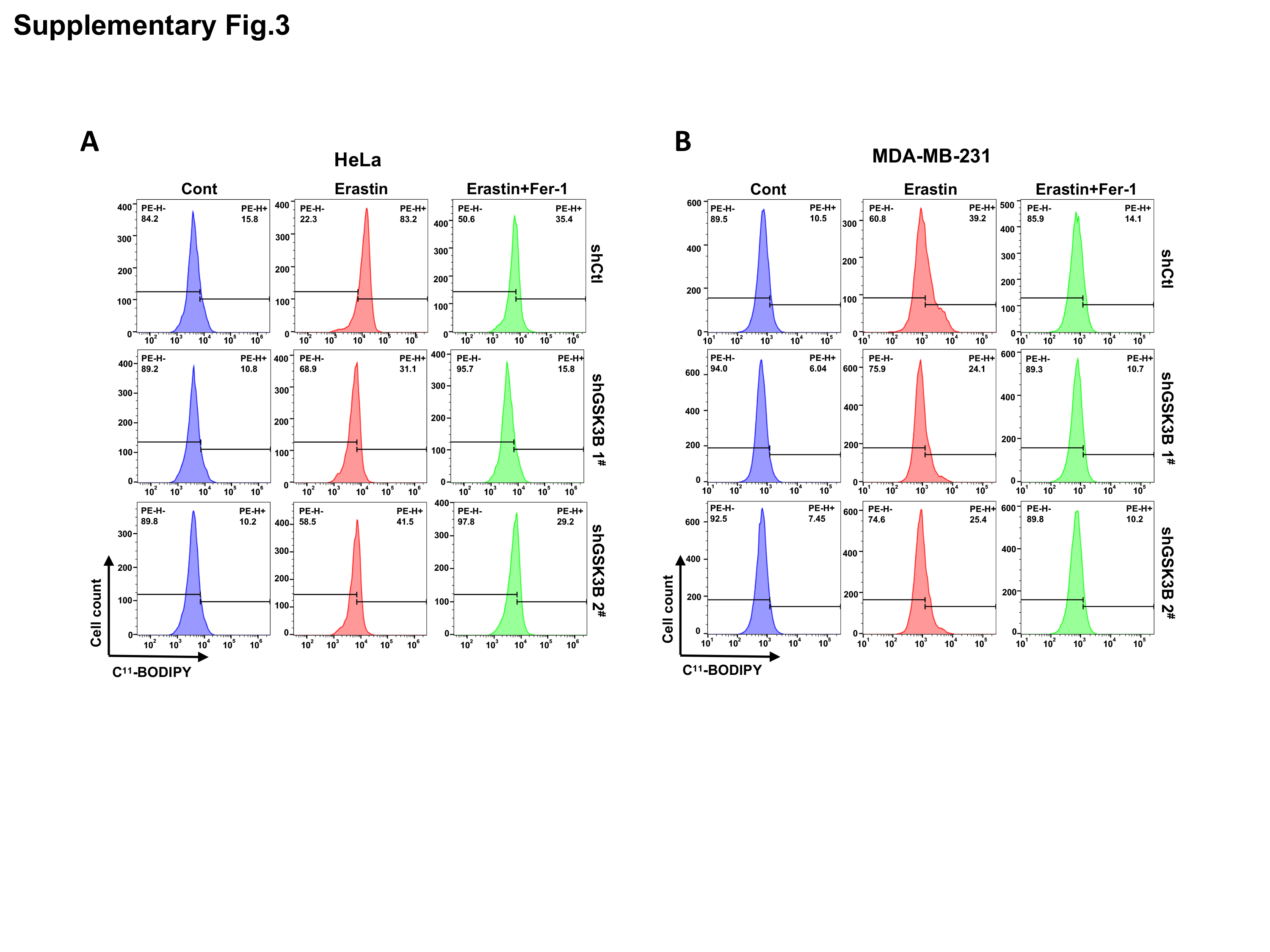

Supplement: Supplementary file 3 — Supplementary Fig.3 [file 41420_2021_726_MOESM3_ESM.tif]

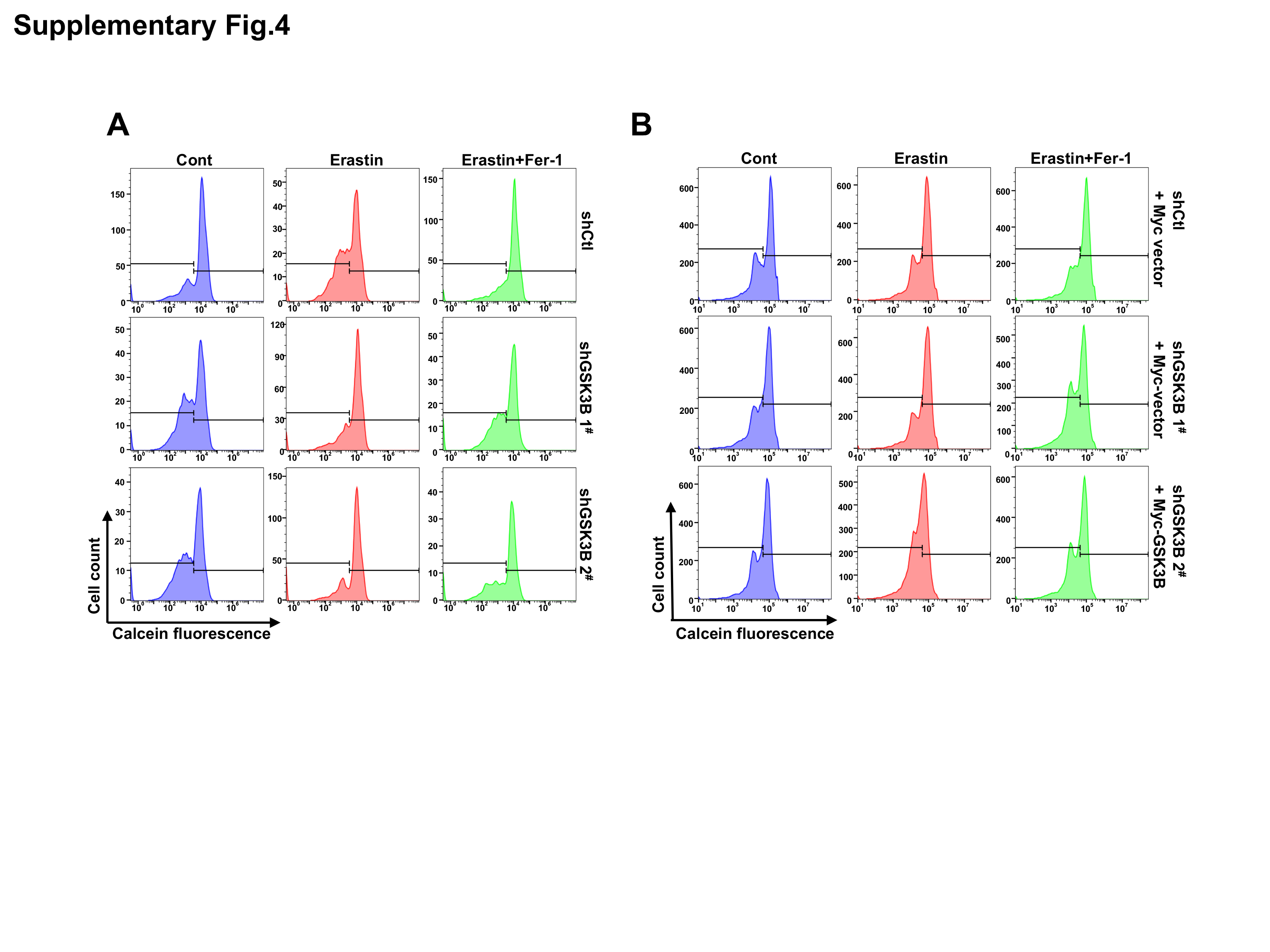

Supplement: Supplementary file 4 — Supplementary Fig.4 [file 41420_2021_726_MOESM4_ESM.tif]

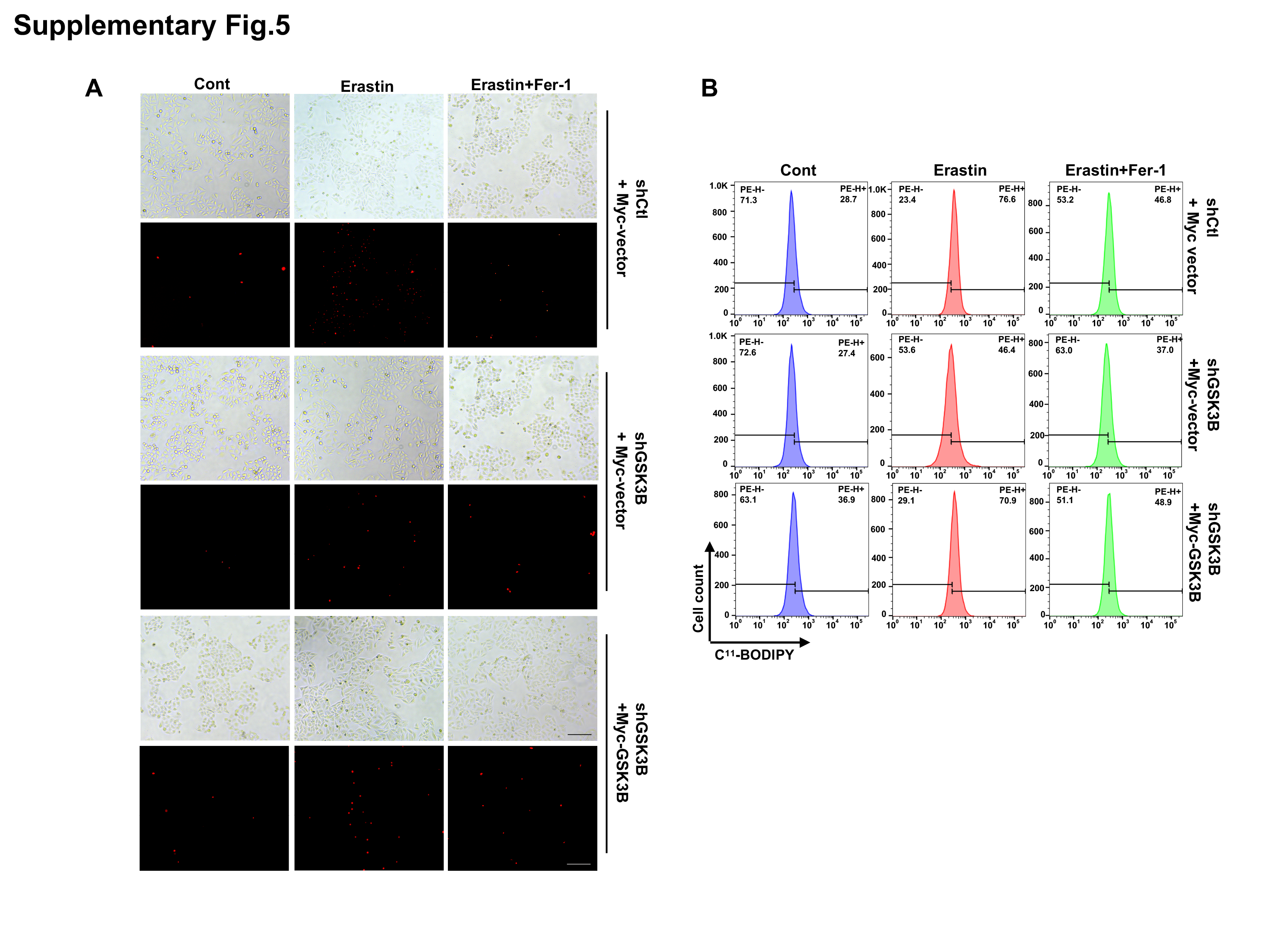

Supplement: Supplementary file 5 — Supplementary Fig.5 [file 41420_2021_726_MOESM5_ESM.tif]
