# Supplementary material for: GSK-3β manipulates ferroptosis sensitivity by dominating iron homeostasis
Source: Cell Death Discov. 2021 Nov 3;7:334. doi: 10.1038/s41420-021-00726-3 (PMC8566589; doi:10.1038/s41420-021-00726-3)
Supplement: Supplementary file 6 — Supplementary Figure legends [file 41420_2021_726_MOESM6_ESM.docx]

**Supplementary Figures**

**Supplementary Fig. 1 Inhibition of GSK3 kinase activity had no effect on GSH and GSSH level.**

Indicated HeLa cells were treated with erastin (35μM) for 24 h in combination with or without Fer-1 (20μM) and LY (20nM), and intracellular GSH **(A)** and GSSG **(B)** level were examined by the Glutathione Assay Kit. Data shown represent mean ± SD from three independent experiments. Comparisons were made using Student’s t-test; *p < 0.05, **p < 0.01, ***p < 0.001.

**Supplementary Fig. 2 GSK3B knockdown suppressed erastin-induced cell death**

**(A)** Indicated HeLa cells were treated with erastin (35 μM) and Fer-1 (20 μM) for 24h, and cell death was measured by propidium iodide (PI) staining using a flow cytometry.

**(B)** Indicated MDA-MB-231 cells were treated with erastin (40 μM) and Fer-1 (20 μM) for 24h, and cell death was measured by propidium iodide (PI) staining using a flow cytometry.

**(C)** Indicated HeLa cells were treated with erastin (35 μM) and Fer-1 (20 μM) for 24h, and cell death was measured by propidium iodide (PI) staining using fluorescence microscopy. Shown is a representative image from three independent experiments.

**Supplementary Fig. 3 GSK3 inhibition or GSK3B knockdown affect accumulation of lipid ROS.**

**(A)** Indicated HeLa cells were treated with erastin (35 μM) with or without Fer-1(20 μM) or LY (20 nM) for 24h, and lipid ROS production was detected by flow cytometry using the fluorescent probe C^11^-BODIPY.

**(B)** Indicated MDA-MB-231 cells were treated with erastin (45 μM) with or without Fer-1(20 μM) or LY (20 nM) for 24h, and lipid ROS production was detected by flow cytometry using C^11^-BODIPY. Shown is a representative image from three independent experiments.

**Supplementary Fig. 4 GSK-3β modulates labile iron pool level** **induced by erastin**

(A) Indicated shCtl, shGSK3B 1^#^ and 2^#^ HeLa cells were treated with erastin (35μM) with or without Fer-1(20μM) for 24h, and intracellular labile iron level was assayed by flow cytometry using Calcein-AM probe.

**(B)** Indicated shCtl or shGSK3B HeLa cells were transfected with either a control plasmid (Myc-vector) or Myc-GSK3B plasmid. shCtl HeLa cells transfected with Myc-vector, shGSK3B and rescued GSK3B cells were treated with erastin (35 μM) with or without Fer-1 (20 μM) for 24 h, and intracellular labile iron level was assayed by flow cytometry using Calcein-AM probe. Shown is a representative image from three independent experiments.

**Supplementary Fig. 5 Re-expression of GSK-3β restores GSK3B depletion-resisted ferroptosis.**

**(A)** shCtl or shGSK3B HeLa cells transfected with Myc-vector or Myc-GSK3B were treated with erastin (35 μM) with or without Fer-1 (20 μM) for 24 h, and cell death was measured by propidium iodide (PI) staining using fluorescence microscopy. Upper, bright field; Down, PI signal. Scale bar, 100 μm.

**(B)** shCtl or shGSK3B HeLa cells transfected with Myc-vector or Myc-GSK3B were treated with erastin (35 μM) with or without Fer-1 (20 μM) for 24 h, and lipid ROS production was detected by flow cytometry using the fluorescent probe C^11^-BODIPY. Shown is a representative image from three independent experiments.
